# Updated Insights into 3D Architecture Electrodes for Micropower Sources

**DOI:** 10.1002/adma.202103304

**Published:** 2021-09-24

**Authors:** Mo Sha, Huaping Zhao, Yong Lei

**Affiliations:** ^1^ Fachgebiet Angewandte Nanophysik Institut für Physik & IMN MacroNano Technische Universität Ilmenau 98693 Ilmenau Germany

**Keywords:** 3D architecture electrodes, energy and power density, lifespan, microbatteries, microsupercapacitors

## Abstract

Microbatteries (MBs) and microsupercapacitors (MSCs) are primary on‐chip micropower sources that drive autonomous and stand‐alone microelectronic devices for implementation of the Internet of Things (IoT). However, the performance of conventional MBs and MSCs is restricted by their 2D thin‐film electrode design, and these devices struggle to satisfy the increasing IoT energy demands for high energy density, high power density, and long lifespan. The energy densities of MBs and MSCs can be improved significantly through adoption of a 2D thick‐film electrode design; however, their power densities and lifespans deteriorate with increased electrode thickness. In contrast, 3D architecture electrodes offer remarkable opportunities to simultaneously improve MB and MSC energy density, power density, and lifespan. To date, various 3D architecture electrodes have been designed, fabricated, and investigated for MBs and MSCs. This review provides an update on the principal superiorities of 3D architecture electrodes over 2D thick‐film electrodes in the context of improved MB and MSC energy density, power density, and lifespan. In addition, the most recent and representative progress in 3D architecture electrode development for MBs and MSCs is highlighted. Finally, present challenges are discussed and key perspectives for future research in this field are outlined.

## Graphical abstract

3D architecture electrode design enhances energy storage capability within a limited footprint area. The superiority of 3D architecture electrodes for performance improvement of microbatteries and microsupercapacitors is comprehensively addressed. The most recent research advances, current challenges, and future perspectives are outlined and discussed.

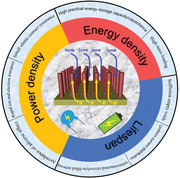

## 1 Introduction

Considerable growth and innovation in the fields of microelectronics and microsystems have triggered the advent of the Internet of Things (IoT), and IoT‐based applications are currently being adopted in almost every area of human life.^[^ ^1^, ^2^ ^]^ Ideally, a complete IoT system integrates multiple types of sensor and actuator with microdevices for data storage, processing, and wireless transmission, and all of these components work together to form a cohesive ecosystem. Moreover, this system should be capable of operating off‐grid and maintenance‐free. Therefore, a functioning IoT system requires high‐performance and miniaturized (with a small footprint of less than 1 cm^2^) power sources, which i) provide sufficient energy for operation of all IoT‐system components for extended periods of time; ii) satisfy instantaneous high energy requirements for wireless data transmission; iii) reduce power‐source maintenance and replacement cycles; and iv) possess dimensional compatibility for integration with other small‐scale microdevices.^[^ ^3^, ^4^, ^5^, ^6^ ^]^ However, no current micropower sources simultaneously possess all four key features, and this problem is hindering successful implementation of IoT‐based applications. Hence, successful development of micropower sources with dimensional compatibility, high energy, high power, and long lifespan is essential for further advancement of IoT‐based applications.

**Figure 1:**
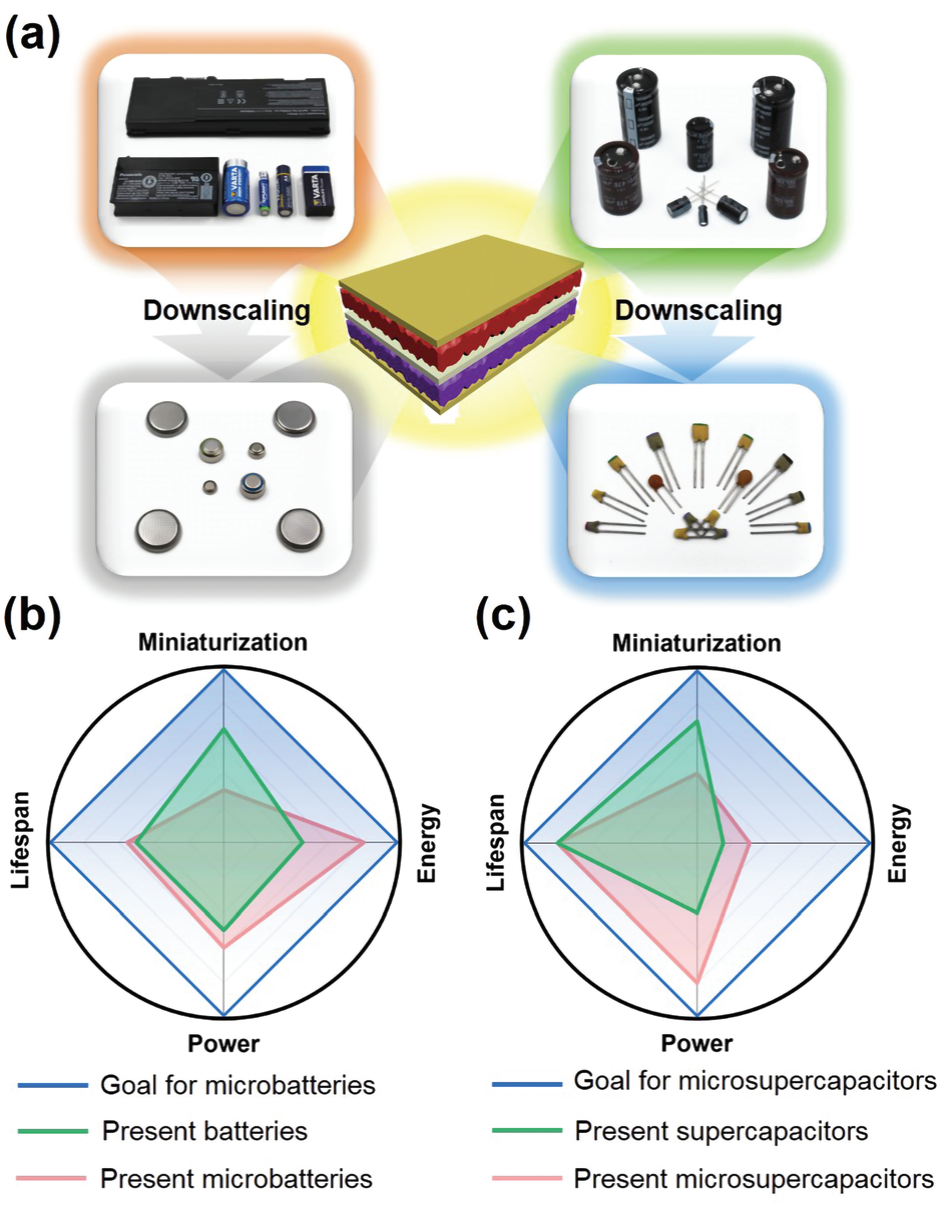
a) Schematic illustration of MB and MSC fabrication through downscaling of their corresponding bulky counterparts. Radar charts of important target metrics for b) MBs and c) MSCs as micropower sources for IoT devices.

Currently, the major IoT‐device micropower sources on the market are microbatteries (MBs) and microsupercapacitors (MSCs).^[^ ^7^, ^8^, ^9^, ^10^, ^11^, ^12^, ^13^ ^]^ Commercial MBs and MSCs are essentially microscale versions of conventional batteries and supercapacitors, respectively. Similar to their bulky counterparts, they usually consist of laminated 2D thin‐film electrodes and are produced using a similar downscaling technique, as shown in **Figure** **1a**. Thus, the mature manufacturing process for conventional batteries and supercapacitors can be employed to produce MBs and MSCs in a cost‐effective manner. However, a key challenge for MBs and MSCs is the decrease in attainable energy and power that coincides with dimensional downscaling (Figure 1b,c). Normally, the delivered energy and power of conventional batteries and supercapacitors are subject to their dimensions, with larger dimensions corresponding to higher values. As the practically available energy and power of commercial MBs and MSCs are also dimension‐dependent, these properties are severely restricted by the small device dimensions.^[^ ^14^, ^15^, ^16^ ^]^

Owing to the 2D geometry limits of thin‐film electrodes, simultaneous enhancement of the energy and power available for the limited device footprint (i.e., the areal energy and power densities) has been considered impossible. To overcome this limitation, the concept of 3D MBs and MSCs has emerged, in which 3D architecture electrodes are used for MB and MSC fabrication.^[^ ^17^, ^18^, ^19^, ^20^, ^21^, ^22^, ^23^, ^24^, ^25^, ^26^ ^]^ To date, various 3D micro‐/nanostructures of different materials and with different morphologies have been produced and extensively studied as advanced electrodes for improving the energy storage performance of conventional batteries and supercapacitors.^[^ ^27^, ^28^, ^29^, ^30^ ^]^ The basic design concept is to use the third dimension (i.e., height) of the electrode to load more active materials, with short ion transport distances between electrodes being maintained despite the increased electrode thickness. In this structure, the improved areal energy density does not compromise the areal power density. Inspired by the performance improvements achieved for conventional batteries and supercapacitors using 3D architecture electrodes, considerable research attention has been focused on 3D MBs and MSCs in the past decade, with the aim of developing micropower sources to satiate both dimensional and energetic requirements for on‐chip integration. In particular, remarkable progress has been achieved in the design and fabrication of 3D architecture electrodes for 3D MB and MSC construction.^[^ ^31^, ^32^, ^33^, ^34^, ^35^ ^]^ Through top‐down and/or bottom‐up fabrication techniques (such as templating, self‐assembly, lithography, and 3D printing), various 3D architecture electrodes have been rationally designed and produced, including ensembles of micro‐/nanostructures (e.g., wire, rod, tube, sheet, and pore structures), hierarchical structures, and 3D porous structures.^[^ ^12^, ^33^ ^]^ As expected, these 3D architecture electrodes enable large specific surface areas and short ion transport distances and, therefore, hold promise for realization of 3D MBs and MSCs with high energy and power capabilities. However, despite the continuous innovations and advancements in the design and fabrication of 3D architecture electrodes, 3D MBs and MSCs based on this technology have not yet been commercialized, apart from some proof‐of‐concept examples.^[^ ^7^, ^8^, ^21^, ^22^, ^23^, ^24^, ^25^, ^26^, ^27^, ^28^, ^29^, ^30^, ^31^, ^32^, ^33^, ^34^, ^35^, ^36^ ^]^

This review presents a comprehensive overview of the most recent advances in the design, fabrication, and employment of advanced 3D architecture electrodes for improved MB and MSC energy, power, and lifespan. The aim is to further understanding of the superiority of 3D architecture electrodes over 2D thick‐film electrodes for MBs and MSCs, and to provide an update on the main challenges associated with 3D architecture electrodes themselves as well as their assembly into 3D MBs and MSCs. In addition, potential solutions to these challenges are discussed and key perspectives for future research in this field are outlined.

The remainder of this paper is structured as follows. Section 2 elucidates the superiority of the 3D architecture electrode design compared to that of the 2D thick‐film electrode, while Sections 3 and 4 discuss state‐of‐the‐art 3D architected electrodes for MBs and MSCs, respectively. Finally, Section 5 considers existing research challenges and future perspectives.

## 2 Superiority of 3D Architecture Electrode over 2D Thick‐Film Electrode

**Figure 2:**
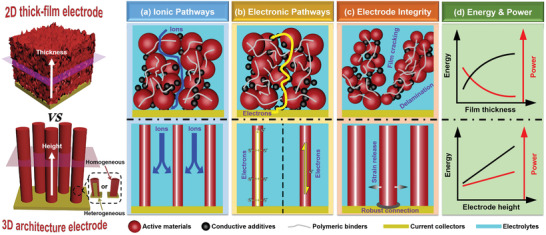
Schematics comparing a–c) ionic pathways, electronic pathways, and electrode integrity, respectively, of 2D thick‐film electrode (top) and 3D architecture electrode (bottom); and d) comparison of energy and power trends with increasing film thickness and electrode height for the 2D thick‐film electrode and 3D architecture electrode.

As demonstrated above, the space constraints of microsystems and on‐chip electronics for IoT‐based applications hinder realization of integrated micropower sources based on conventional MBs and MSCs and having sufficient energy and power levels. For 2D thin‐film electrode configurations of conventional MBs and MSCs, in which the electrode materials (e.g., electrochemically active materials, polymeric binders, and conductive additives) are slurries/pastes coated on planar metallic current collectors (i.e., copper (Cu) and aluminum (Al) foils), the general method toward achieving higher areal energy densities involves fabrication of compact and dense electrodes.^[^ ^37^, ^38^ ^]^ The thicker the electrode, the higher the electrochemically active material content and the higher the areal energy density. However, a series of passive problems arise for the 2D thick‐film electrode design, including sluggish reaction kinetics, tortuous charge (electrons and ions) transfer pathways, and weak mechanical integrity. Both sluggish kinetics and tortuous charge transfer pathways deteriorate power performance, while weak mechanical integrity yields poor lifespan.^[^ ^38^ ^]^ In contrast to the 2D thick‐film electrode design, the 3D architecture electrode design has remarkable advantages with regard to the ionic pathways, electronic pathways, and electrode integrity, as shown in **Figure** **2**; hence, this design offers considerable opportunities for simultaneous improvement of the areal energy and power densities of both MBs and MSCs, while also prolonging their lifespans.

### 2.1 Areal Energy Density Enhancement via 3D Architecture Electrode Design

The areal energy density of both MBs and MSCs is proportional to the mass loading (mg cm^–2^) of the electrochemically active materials; thus, an effective approach to increasing the areal energy density is to load more electrochemically active materials within the constrained footprint area. Unlike the 2D thick‐film electrode design, in which the electrode thickness is increased, the 3D architecture electrode design utilizes the third dimension, that is, height, to increase the mass loading of the electrochemically active materials.

3D architecture electrodes can be categorized as homogeneous or heterogeneous based on their configuration.^[^ ^31^ ^]^ For homogeneous 3D architecture electrodes, the electrochemically active materials are directly synthesized into various 3D architectures on a current collector. For heterogeneous 3D architecture electrodes, a current collector is first fabricated in the 3D architecture and a thin layer of electrochemically active materials is then conformally deposited; thus, these as‐fabricated electrodes have a core‐shell structure. For both homogeneous and heterogeneous 3D architecture electrodes, the mass loading of the electrochemically active materials and, hence, the areal energy density, can be increased by simply increasing the electrode height.^[^ ^22^, ^23^, ^24^ ^]^ In addition to enhanced mass loading, 3D architecture electrodes provide a large surface area and improved electrolyte percolation. These features can facilitate the electrochemically active materials and, hence, maximize their practical energy storage capacity/capacitance. Thus, performance close to the theoretical specific capacity/capacitance may be obtained. Overall, for MBs and MSCs consisting of 3D architecture electrodes, synchronous enhancement of both the mass loading and practical capability/capacitance of the electrochemically active materials increases the areal energy density.

### 2.2 Areal Power Density Enhancement via 3D Architecture Electrode Design

In conventional 2D MBs and MSCs, the areal energy and power densities are strongly coupled, which induces an inevitable trade‐off between the attainable energy and power.^[^ ^23^, ^24^, ^25^, ^30^, ^31^, ^32^ ^]^ Fast and efficient charge (ion and electron) transport within the electrodes is a key prerequisite for high‐power performance. In 2D thin‐ and thick‐film electrodes, random and close packing of electrochemically active materials, polymeric binders, and conductive additives generates tortuous ion transport pathways that elongate the ion diffusion length (see Figure 2). The thicker the electrode, the more tortuous the ion transport pathway and the lower the ion diffusion efficiency. Further, the electron transport pathways in 2D thin‐ and thick‐film electrodes are both long and tortuous, being strung together via conductive additives (e.g., carbon blacks) before reaching the current collectors. Moreover, the insulating polymeric binders that are usually necessary for electrode structure stabilization further increase the tortuosity of the electron transport pathways. Thus, ion and electron transport in 2D thick‐film electrodes is becoming increasingly sluggish, and consequently, any expected energy enhancement would be accompanied by an undesirable reduction in power density.

In contrast, the inverse relationship between energy and power can be decoupled for a 3D architecture electrode.^[^ ^23^, ^32^ ^]^ First, both homogeneous and heterogeneous 3D architecture electrodes are free from polymeric binders and conductive additives; thus, the adverse effects of these components on ion and electron transport are avoided. Second, the open porous structure of a 3D architecture electrode permits formation of an interconnected electrolyte‐filled network for rapid ion transport. Third, unlike 2D thick‐film electrodes, an unimpeded channel for rapid ion transport can be retained when the 3D architecture electrode height is increased. Fourth, for heterogeneous 3D architecture electrodes with a thin coating of electrochemically active materials on a 3D architecture current collector, electrons from active sites must only migrate a very short distance to reach the current collector, and vice versa. This maximum migration distance is approximately equal to the coating thickness of the electrochemically active materials, but is independent of the height of the heterogeneous 3D architecture electrode. In the case of a homogeneous 3D architecture electrode, the intrinsic limited conductivities of most of the electrochemically active materials induce a progressive increase in the ohmic resistance with increasing electrode height; however, unlike 2D thick‐film electrodes, the electrochemically active materials can provide directional and convenient electron transport paths. Fifth, in contrast to the laminated structure between the electrode materials and current collectors in 2D thin‐ and thick‐film electrodes, 3D architecture electrodes have an integrated structure of electrochemically active materials and current collectors. This structure minimizes the ohmic contact resistance between the electrochemically active materials and current collectors, especially at high currents.

Owing to these five advantages for ion and electron transport, the 3D architecture electrode design promotes the reaction kinetics occurring during fast charging–discharging processes, thereby enhancing the power performance. Thus, simultaneous enhancement of both the areal energy and power densities can be achieved by simply increasing the electrode height.

### 2.3 Long Lifespan with 3D Architecture Electrode Design

Another major problem affecting 2D thick‐film electrodes is mechanical instability during charging–discharging, which can mainly be ascribed to non‐uniform distribution of internal high stresses in the electrode layer (i.e., the composite layer of electrochemically active materials, polymeric binders, and conductive additives). Generally, high stress generation within 2D thick‐film electrodes results from expansion/volume variation of the electrochemically active materials, gaseous byproducts of the side reactions, the differential concentration profiles of the ions, and/or inhomogeneous current distribution.^[^ ^39^, ^40^, ^41^, ^42^, ^43^, ^44^ ^]^ These stresses often cause mechanical fracturing of the electrodes during charging–discharging, such as, cracking/pulverization of the electrode layer or delamination of this layer from the current collectors, which yields loss of electrical contact. Such mechanical fracturing of the electrodes subsequently induces severe performance degradation and lifespan decline of the MBs and MSCs.

In contrast, the 3D architecture electrode design is an attractive solution that effectively circumvents the adverse effects of these stresses.^[^ ^38^, ^39^ ^]^ First, 3D architecture electrodes have sufficient empty voids that locally accommodate the large expansion/volume variation of the electrochemically active materials upon cycling, thereby alleviating the stress deriving from these severe volume changes. Second, the 3D architecture electrode design eliminates inhomogeneous current distribution by strategically optimizing the structural and morphological parameters. The resulting current distribution homogeneity in the electrode efficiently prevents local polarization and, therefore, reduces the risk of potential side reactions. Third, owing to the integrated and binder‐/additive‐free structure of these electrodes, the delamination issue is not a concern. Fourth, the interconnected electrolyte‐filled network within the 3D architecture electrode helps diminish the electrolyte concentration gradient; this reduction positively influences the ion diffusion kinetics at higher charge/discharge rates, which in turn ensures a high degree of reaction homogeneity to eliminate the local stress concentration in the electrode. The four advantages listed above indicate that the 3D architecture electrode design can circumvent the mechanical instability issue that arises for 2D thick‐film electrodes, thereby achieving long lifespans for MBs and MSCs.

Overall, the 3D architecture electrode design offers the potential for maximizing energy storage capacity within a limited footprint area. Through rational optimization of the structure and morphology of these electrodes, the multiple synergistic effects due to the advantages discussed above can simultaneously enhance the MB and MSC energy and power densities, as well as, their lifespans. This potential, as well as, the increasing pursuit of high‐performance micropower sources, has motivated significant research into the development of advanced 3D architecture electrodes for MBs and MSCs.

## 3 State‐of‐the‐Art 3D Architecture Electrodes for Microbatteries

As apparent from Figure 1b, the energy, power, and lifespan performance of existing MBs must be significantly improved to satisfy the energetic requirements of IoT devices. In the following, we briefly describe representative 3D architecture electrodes for MBs and introduce the associated design principles and fabrication strategies.

**Figure 3:**
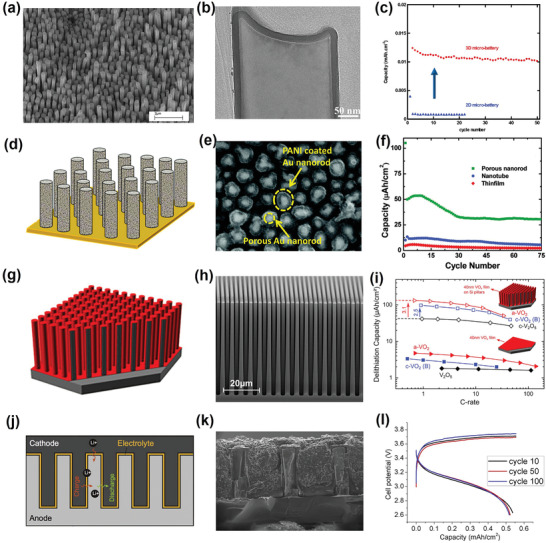
a) Scanning electron microscopy (SEM) image of Al‐nanorod current collectors. b) High‐resolution transmission electron microscopy image of 17‐nm‐thick TiO_2_ layer conformally deposited on Al nanorods. c) Capacity comparison between 3D MBs with TiO_2_ deposited on Al nanorods and 2D MBs with TiO_2_ deposited on flat Al plate. a–c) Reproduced with permission.^[^ ^45^ ^]^ Copyright 2009, Wiley‐VCH. d) Schematic diagram of vertically aligned arrays of PANI‐coated nanoporous Au nanorods. e) SEM image showing PANI and Au components of PANI‐coated nanoporous Au nanorod. f) Cyclability comparison of MBs with three different electrode configurations: planar, nanotube, and nanoporous nanorod. d–f) Reproduced with permission.^[^ ^46^ ^]^ Copyright 2012, American Chemical Society. g) Schematic diagram of VO*_x_*‐coated Si micropillar electrodes as 3D architecture electrodes for MBs. h) Cross‐sectional SEM image of Si micropillar substrate. i) Rate capability comparison of planar Si and Si micropillar electrodes with the same 40‐nm‐thick VO*_x_* layer, including amorphous VO_2_ (a‐VO_2_), crystalline VO_2_ (c‐VO_2_), and crystalline VO_5_ (c‐VO_5_). g–i) Reproduced with permission.^[^ ^59^ ^]^ Copyright 2017, American Chemical Society. j) Schematic of 3D Li‐ion MBs based on partially lithiated Si micropillar arrays as both scaffold and anode and k) corresponding cross‐sectional SEM image. l) Comparison of charging–discharging profiles at 10th, 50th, and 100th cycles indicating excellent cyclability of 3D Li‐ion MBs. j–l) Reproduced with permission.^[^ ^61^ ^]^ Copyright 2018, Elsevier.

The stored energy is directly proportional to the mass of electrochemically active materials; therefore, increasing the electrochemically active material loading within the limited footprint area is an attractive means of increasing the MB energy density. To this end, 3D architecture electrodes fully exploit the third dimension (height) to improve the surface‐to‐volume ratio and allow loading of more electrochemically active materials. Notably, the thin layer of active materials remains unchanged, similar to traditional 2D thin‐film electrodes. For example, Cheah et al. previously reported use of self‐supported Al nanorods coated with a uniform layer of titanium dioxide (TiO_2_) as 3D nanoelectrodes for lithium (Li)‐ion MBs (**Figure** **3a**,b).^[^ ^45^ ^]^ In that work, self‐supported Al nanorods were synthesized as current collectors through anodic aluminum oxide template‐assisted electrodeposition; the active materials (TiO_2_) were then deposited onto the Al nanorods through atomic layer deposition (ALD). Compared to a 2D flat Al substrate, the third dimension (height) allowed the Al nanorods to provide a large surface area with an area gain of ≈10 for loading of active materials. When coated with the same 17‐nm‐thick TiO_2_ layer, the mass loading of the TiO_2_ deposited onto the Al nanorods was 0.0667 mg cm^–2^ compared to the 0.0066‐mg cm^–2^ mass loading achieved for the flat Al substrate. Finally, the areal capacity of the assembled 3D MB was increased by a factor of almost 10 compared to that of 2D MB (Figure 3c).

Similarly, Gowda et al. fabricated nanoporous gold (Au) nanorod arrays as current collectors, which provided an increased surface area for polyaniline (PANI) deposition, as shown in Figure 3d,e.^[^ ^46^ ^]^ Owing to the higher PANI mass loading, the nanoporous Au‐nanorod‐based electrode exhibited a much higher areal capacity than those of planar Au or Au‐nanotube‐based electrodes (Figure 3f). Moreover, benefiting from the open volume (or effective porosity) of these 3D architecture electrodes, the electrolyte ions could rapidly migrate and reach all accessible active sites within the electrodes. Hence, the utilization efficiency of the electrochemically active materials was enhanced and further contributed to the areal energy density improvement. Subsequently, a number of metallic 3D architectures with high specific surface areas were developed to serve as current collectors for 3D architecture electrodes for MBs, so as to elevate the energy density per footprint area.^[^ ^47^, ^48^, ^49^, ^50^, ^51^, ^52^, ^53^ ^]^

In addition to 3D architectures involving metals, vertically ordered silicon (Si) micropillar arrays with high aspect ratios have also been widely studied as scaffolds for preparation of 3D architecture electrodes, owing to the high technical maturity of Si microstructuring.^[^ ^54^, ^55^, ^56^, ^57^, ^58^ ^]^ For instance, Mattelaer et al. utilized Si micropillars with a high surface enhancement factor of 20.6 as a 3D architecture scaffold (Figure 3g); this scaffold was then conformally coated with a 40‐nm‐thick mixed‐valence vanadium oxide (VO*_x_*) layer as the active material via ALD.^[^ ^59^ ^]^ Compared to an electrode consisting of a 40‐nm‐thick VO*_x_* film deposited on a planar Si substrate, the Si‐micropillar‐based electrode demonstrated more than 20‐fold enhancement of the areal capacity (Figure 3h,i). Indeed, Si is well known as a promising anode for Li‐ion batteries, having a high theoretical capacity of up to 4000 mAh g^–1^.^[^ ^60^ ^]^ Undoubtedly, the high capacity of Si is of considerable benefit for improving MB energy density when employed as an anode for Li‐ion MBs. However, fully lithiated Si is required for realization of such a high capacity, but this substance undergoes a volume expansion of ≈300%.^[^ ^60^ ^]^ Considering the small MB volume, it is difficult to provide sufficient free space to accommodate such enormous volume changes, and this requirement hinders application of Si anodes in Li‐ion MBs. In this context, Hur et al. fabricated partially lithiated Si micropillar arrays as both a 3D architecture scaffold and an anode for application in Li‐ion MBs (Figure 3j,k).^[^ ^61^ ^]^ High‐aspect‐ratio Si micropillar arrays were first prepared through conventional dry etching of a Si wafer. Thereafter, the Si micropillar arrays were partially lithiated (to 10% of the theoretical capacity); hence, the volume change was controlled to well within the acceptable limits during charging–discharging. Remarkably, the assembled all‐solid‐state Li‐ion MBs exhibited a high areal energy density of up to 5.2 mWh cm^–2^ (1.8 mAh cm^–2^ areal capacity) and, more impressively, withstood 100 cycles at a high areal energy density of 1.6 mWh cm^–2^ (0.5 mAh cm^–2^ areal capacity), as shown in Figure 3l. The work of Hur et al. constitutes a valuable reference for the design of 3D architecture MB electrodes based on electrochemically active materials that have high theoretical specific capacity but suffer from extremely large volume expansion.^[^ ^61^ ^]^

**Figure 4:**
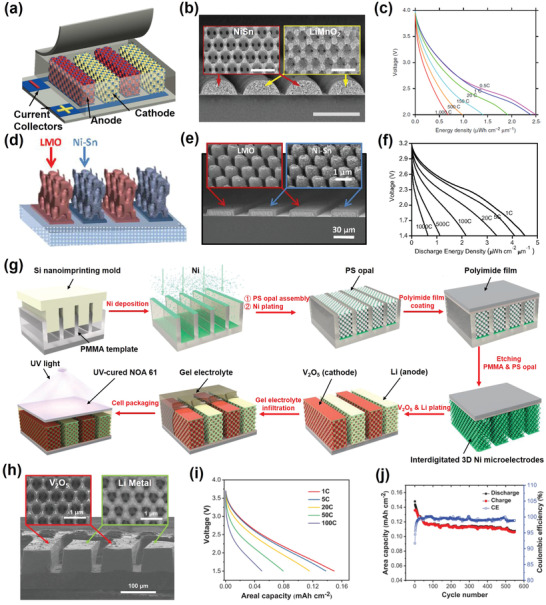
a) Schematic showing Li‐ion MBs with interdigital patterns of 3D bicontinuous nanoporous NiSn anodes and LiMnO_2_ cathodes. b) Cross‐sectional SEM image of NiSn anode and LiMnO_2_ cathode. c) Discharge profiles of MBs with interdigital patterns of 3D bicontinuous nanoporous NiSn anodes and LiMnO_2_ cathodes at rates ranging from 0.5 to 1000 C. a–c) Reproduced with permission.^[^ ^62^ ^]^ Copyright 2013, Nature Publishing Group. d) Schematic illustration of MBs with NiSn anodes and LiMnO_2_ cathodes, both having 3D holographic structures. e) Cross‐sectional SEM image of NiSn anode and LiMnO_2_ (LMO) cathode. f) Discharge voltage profiles of MBs at rates ranging from 1 to 1000 C. d–f) Reproduced with permission.^[^ ^63^ ^]^ Copyright 2015, National Academy of Sciences. g) MB fabrication scheme combining imprint lithography, colloidal templating, and electrodeposition; PMMA: Poly(methyl methacrylate), PS: Polystyrene, UV: Ultraviolet. h) Cross‐sectional SEM image of interdigitated vanadium pentoxide (V_2_O_5_) cathode and Li metal anode, both having an inverse opal structure. i) Discharge voltage profiles of V_2_O_5_//Li MBs at rates from 1 to 100 C. j) Cycling performance of V_2_O_5_/Li MBs at 1‐C charging–discharging rate. g–j) Reproduced with permission.^[^ ^64^ ^]^ Copyright 2021, Wiley‐VCH.

Owing to the open volume of the 3D architecture electrode design, the interconnected electrolyte‐filled network facilitates fast transport of electrolyte ions in the electrodes and, simultaneously, avoids local depletion of electrolyte ions, especially at high rates; these characteristics permit fast charging and discharging. Furthermore, establishment of a convenient electron transport pathway in the 3D architecture electrode decreases the electrical resistance of the entire electrode compared to 2D thick‐film electrodes (see Figure 2 and Section 2.2). Considering these properties, Pikul et al. designed high‐power‐density and high‐energy‐density Li‐ion MBs based on 3D bicontinuous nanoporous electrodes (**Figure** **4a**).^[^ ^62^ ^]^ As depicted in Figure 4b, nickel–tin (NiSn) anodes and lithiated manganese oxide (LiMnO_2_) cathodes were separately and conformally coated onto interdigitated, highly porous Ni scaffolds. In that process, the highly porous Ni scaffolds were synthesized via a colloidal templating strategy. The resultant electrode design featured short electron and ion transport pathways, yielding high power density while maintaining high mass loading of the active material to achieve high energy density. From Figure 4b, the as‐fabricated MBs exhibited excellent rate capability at discharge rates of 0.5–1000 C. Impressively, the optimized MBs delivered a high power density of up to 7.4 mW cm^–2^ μm^–1^ at an ultrahigh rate of 870 C and a high energy density of up to 15 μWh cm^–2^ μm^–1^ at 1.5 C.

Considering the complexity and limitations of the colloidal templating strategy for large‐scale production, Ning et al. developed an on‐chip compatible technique by combining 3D holographic lithography with conventional photolithography to fabricate 3D architecture MB electrodes (Figure 4d,e).^[^ ^63^ ^]^ Similar to the aforementioned interdigitated, highly porous Ni scaffolds, the resultant interdigitated 3D holographic Ni current collectors with periodically mesostructured lattices had a similar structural design concept; that is, efficient electron pathways, short ion diffusion lengths, and an interconnected ion transport network. However, the preparation procedures were highly compatible with commercialized microelectronic processing, rendering mass production feasible. For the same NiSn anode and LiMnO_2_ cathode used by Pikul et al., the resultant Li‐ion MBs exhibited both high areal energy and power densities through optimization of the interdigitated electrode spacing.^[^ ^62^ ^]^ Furthermore, more than 80% of the initial capacity was retained after 100 continuous cycles at various rates.

Most recently, Sun et al. combined imprint lithography, colloidal templating, and electrodeposition to fabricate thick 3D architecture electrodes with a high active volume fraction; hence, they realized high mass loading of the electrochemically active materials while simultaneously retaining fast ion and electron transport kinetics.^[^ ^64^ ^]^ Figure 4g is a schematic of the MB fabrication process. First, interdigitated Ni inverse opal structures were fabricated as current collectors through a combination of imprint lithography, colloidal templating, and electrodeposition; then, vanadium pentoxide (V_2_O_5_) and Li were deposited as the cathode and anode, respectively (Figure 4h). Thereafter, the electrodes were infilled with a gel electrolyte via capillary force‐guided solvent filling. Finally, the MBs were packaged with a cured photopolymer (Norland optical adhesive 61). The packaged MBs exhibited a supercapacitor‐like peak power density of 75.5 mW cm^–2^ at a high rate of 100 C (Figure 4i), a high peak energy density of 1.242 J cm^–2^, and excellent cyclability, retaining 75% of their initial discharge capacity after 550 or 200 cycles under argon or air, respectively (Figure 4j). The excellent cyclability can also be attributed to the superiority of 3D architecture electrodes over 2D thick‐film electrodes (see Figure 2 and Section 2.3). For a microscale autonomous device consuming 5 µW in standby mode (100‐s standby time) and 5 mW during data transmission (10‐ms transmit time), MBs are estimated to be capable of supplying power for ≈132 days.^[^ ^64^ ^]^ These results highlight the strong potential of 3D architecture electrodes for further development of high‐performance MBs to advance IoT‐based applications.

**Figure 5:**
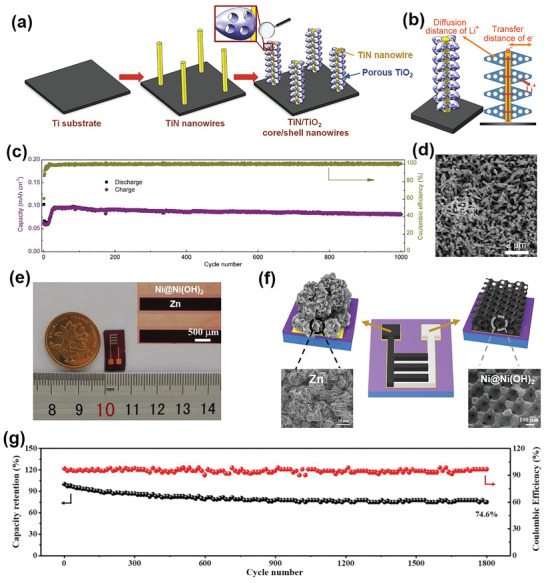
a) Schematics of TiN@TiO_2_ coaxial nanowire array fabrication process. b) Schematic diagram illustrating short Li ion diffusion and electron transfer distances in TiN@TiO_2_ coaxial nanowire array electrodes. c) Cycling performance and corresponding coulombic efficiency of TiN@TiO_2_ coaxial nanowire array electrodes. d) SEM images of TiN@TiO_2_ coaxial nanowire array electrodes after 1000 charging–discharging cycles. a–d) Reproduced with permission.^[^ ^65^ ^]^ Copyright 2017, Elsevier. e) Optical images of on‐chip Ni—Zn MBs and corresponding interdigitated electrode. f) Schematic illustration of on‐chip Ni—Zn MBs with hierarchical ordered porous Ni@Ni(OH)_2_ cathode and Zn anode. g) Long‐term cycling stability of on‐chip Ni—Zn MBs. e–g) Reproduced with permission.^[^ ^66^ ^]^ Copyright 2019, Wiley‐VCH.

As previously mentioned in Section 2.3, the 3D architecture electrode design is conducive to MB lifespan improvement by circumventing the adverse stresses arising from severe volume changes of the electrochemically active materials, gaseous byproducts of the side reactions, the differential concentration profiles of the ions, and/or inhomogeneous current distribution. By optimizing the morphologies and structural parameters of 3D architecture electrodes, a variety of proof‐of‐concept MBs showing superior stability for hundreds or even thousands of cycles have been developed.^[^ ^67^, ^68^, ^69^, ^70^, ^71^ ^]^ For example, Wen et al. reported Li‐ion MBs with free‐standing titanium nitride (TiN)@titanium dioxide (TiO_2_) coaxial nanowire arrays as the anode.^[^ ^65^ ^]^ As shown in **Figure** **5a**, free‐standing TiN nanowire arrays were first grown on a Ti substrate to function as nanostructured current collectors; these arrays were then coated with nanoporous anatase TiO_2_ mesocrystals. Figure 5b schematically demonstrates that such an electrode design can simultaneously reduce the electron transport distance and the Li‐ion diffusion distance to yield Li‐ion MBs with excellent energy and power performance. During the lithiation/delithiation process, the void spaces of the TiN@TiO_2_ coaxial nanowires and the nanoporous structure of the TiO_2_ accommodated the volume changes, and the monolithic structure of the TiN@TiO_2_ coaxial nanowires (directly grown on the substrate) helped maintain the structural integrity of the entire electrode. Finally, outstanding cyclability of up to 1000 cycles was realized (Figure 5c), and the TiN@TiO_2_ coaxial nanowire arrays retained their well‐defined, free‐standing morphologies after 1000 lithiation/delithiation cycles (Figure 5d).

In another example, a hierarchically ordered porous Ni@nickel hydroxide (Ni(OH)_2_) electrode was designed and fabricated as a cathode for a quasi‐solid‐state on‐chip Ni—Zn MB with an in‐plane interdigitated electrode structure (Figure 5e,f).^[^ ^66^ ^]^ Benefiting from the interconnected ordered macropore–mesopore network in the Ni@Ni(OH)_2_ electrode, excellent energy and power densities (0.26 mWh cm^–2^ and 33.8 mW cm^–2^, respectively) were simultaneously achieved. More impressively, the Ni—Zn MBs exhibited prominent long‐term stability with 74.6% capacity retention after 1800 charging–discharging cycles (Figure 5g). If MBs with such long lifespans and high energy/power densities were employed as IoT‐device power sources, the MB replacement cycles would be reduced significantly and maintenance‐free operation is accomplished.

## 4 State‐of‐the‐Art 3D Architecture Electrodes for Microsupercapacitors

High power density and an ultra‐long cycle life are two principal advantages of MSCs over MBs; thus, MSCs are suitable for applications with high power requirements. Indeed, MSCs have considerable potential to become the primary micropower sources once their energy density is improved to that of MBs without sacrifice of their high power density and ultra‐long cycle life. Similar to conventional supercapacitors, MSC charge storage occurs via fast and surface‐confined processes at the electrode–electrolyte interface, which can be electrostatic or faradic in nature. Accordingly, enlarging the electrode–electrolyte interface area is an efficient means of improving MSC energy density. As noted in Section 2, a 3D architecture electrode possesses a high specific surface area and its open porous structure permits formation of an interconnected electrolyte‐filled network. The latter not only generates a larger electrode–electrolyte interface area, but also facilitates rapid ion diffusion, which simultaneously enhances the energy and power density. Hence, a large variety of 3D architecture electrodes have been developed and investigated to address the limited energy densities of current MSCs.^[^ ^72^, ^73^, ^74^, ^75^, ^76^, ^77^, ^78^, ^79^ ^]^

**Figure 6:**
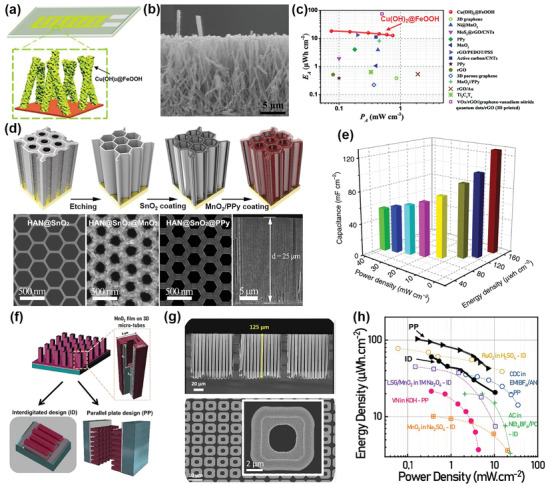
a) Schematic illustration of interdigitated Cu(OH)_2_@FeOOH nanotube‐array electrodes. b) Cross‐sectional SEM image of Cu(OH)_2_@FeOOH nanotube arrays grown on Cu substrate. c) Ragone plot for Cu(OH)_2_@FeOOH‐based in‐plane MSCs compared with other reported in‐plane MSCs. a–c) Reproduced with permission.^[^ ^80^ ^]^ Copyright 2019, Royal Society of Chemistry. d) Illustration of HAN‐based electrode fabrication process and corresponding SEM images of HAN@SnO_2_, HAN@SnO_2_@MnO_2_, and HAN@SnO_2_@PPy electrodes. e) Areal energy and power densities of HAN@SnO_2_@MnO_2_//HAN@SnO_2_@PPy asymmetric MSCs measured at different current densities. d,e) Reproduced with permission.^[^ ^81^ ^]^ Copyright 2020, Nature Publishing Group. f) Schematic representation of MnO_2_‐coated Si microtube electrodes for on‐chip MSCs with interdigitated and parallel plate configurations. g) Cross‐sectional SEM image of Si microtubes and top‐view SEM image of Si microtubes following coating with 580‐nm‐thick layer of MnO_2_. h) Performance comparison of MSCs based on MnO_2_‐coated Si microtube electrodes with reported MSCs tested in aqueous and organic electrolytes. f–h) Reproduced with permission.^[^ ^82^ ^]^ Copyright 2021, Elsevier.

In the past three years in particular, some developed MSCs have benefited from the properties of 3D architecture electrodes to realize battery‐like energy density while retaining high power density and a long cycle life. For example, Xie et al. reported flexible, in‐plane MSCs based on interdigitated copper hydroxide (Cu(OH)_2_)@ferric oxyhydroxide (FeOOH) nanotube array electrodes (**Figure** **6a**).^[^ ^80^ ^]^ Vertically aligned Cu(OH)_2_@FeOOH nanotubes with heights of up to 14 μm were grown directly on interdigitated Cu current collectors (Figure 6b). Nitrogen adsorption/desorption measurements indicated that the Cu(OH)_2_@FeOOH nanotube array electrode had a high specific surface area of 224 m^2^ g^–1^ and a hollow porous structure, which provided abundant electrochemical reactive sites and rapid ion diffusion pathways for fast faradic redox reaction. Hence, the as‐prepared in‐plane MSCs exhibited high areal energy densities of up to 18.07 μWh cm^–2^; this result is among the best reported for in‐plane MSCs (Figure 6c).

In another work, Lei et al. designed 3D architecture electrodes based on a honeycomb alumina nanoscaffold (HAN) for assembly of MSCs with a conventional sandwich structure.^[^ ^81^ ^]^ A robust HAN with ultrahigh cell density and an ultrathin nanoscaffold was first coated with tin oxide (SnO_2_).Then, either magnesium oxide (MnO_2_) or polypyrrole (PPy) was deposited to yield HAN@SnO_2_@MnO_2_ or HAN@SnO_2_@PPy electrodes, respectively (Figure 6d). These electrodes possessed vertically aligned and highly stable nanoporous structures with no aspect ratio limit. Thus, the effects of both the effective ion migration and ample electroactive surface within the limited footprint were maximally synergized, enabling high and reversible capacitive behavior even at high charging–discharging rates and guaranteeing high energy and power performance for the resultant MSCs. The peak energy density of the prepared HAN@SnO_2_@MnO_2_//HAN@SnO_2_@PPy asymmetric MSCs with conventional sandwich structures reached 160 μWh cm^–2^, while a high peak power density of 40 mW cm^–2^ was maintained (Figure 6e). Moreover, the asymmetric MSCs retained 87% of their original capacity after 30 000 charging–discharging cycles.

Similarly, Bounor et al. employed Si microtubes with an area enhancement factor of 47 in a 3D scaffold for construction of 3D architecture electrodes for both in‐plane and sandwich MSCs (Figure 6f).^[^ ^82^ ^]^ The Si microtube length reached 125 μm and the tubular structure was maintained after conformal coating with a 580‐nm‐thick MnO_2_ layer (Figure 6g). The energy and power densities of the in‐plane and sandwich MSCs based on these MnO_2_‐coated Si microtube electrodes were 0.05–0.1 mWh cm^–2^ and 1 mW cm^–2^, respectively; these are some of the highest reported energy/power trade‐off values for state‐of‐the‐art MSCs tested in liquid electrolytes (Figure 6h).

**Figure 7:**
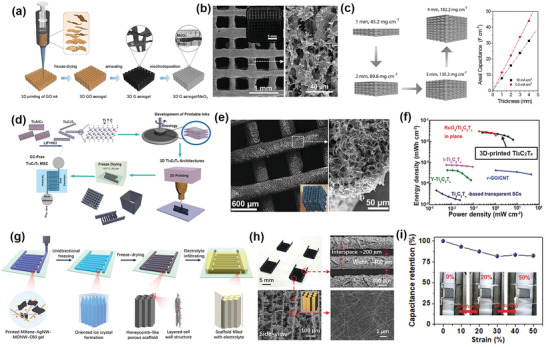
a) Schematic illustration of 3D‐printed graphene (G) aerogel/MnO_2_ electrode fabrication. b) SEM image of 3D‐printed G aerogel lattice. c) Schematics of 3D‐printed G aerogel/MnO_2_ electrodes with different MnO_2_ thicknesses and mass loadings and their corresponding areal capacitance. a–c) Reproduced with permission.^[^ ^83^ ^]^ Copyright 2019, Elsevier. d) Schematic representation of 3D‐printed MSC fabrication with Ti_3_C_2_T*_x_*‐based printable inks. e) SEM images and optical photographs (inset) of 3D‐printed Ti_3_C_2_T*_x_* microlattice. f) Ragone plots of 3D‐printed Ti_3_C_2_T*_x_* MSCs compared with Ti_3_C_2_T*_x_*‐based MSCs fabricated via other techniques. d–f) Reproduced with permission.^[^ ^84^ ^]^ Copyright 2019, Wiley‐VCH. g) Illustration showing fabrication of 3D‐printed electrodes with honeycomb‐like microporous scaffold. h) Optical and SEM images of as‐fabricated 3D‐printed electrodes showing honeycomb‐like micropores. i) Optical images of 3D‐printed electrodes with honeycomb‐like microporous scaffold under stretching from 0% to 50% and corresponding capacitance retention at different stretching ratios. g–i) Reproduced with permission.^[^ ^85^ ^]^ Copyright 2020, Wiley‐VCH.

As an emerging print‐on‐demand technology, 3D printing has been shown to cost‐effectively produce 3D architectures with complex shapes or geometries for various applications, including batteries and supercapacitors.^[^ ^86^, ^87^, ^88^, ^89^, ^90^, ^91^, ^92^ ^]^ Recently, 3D architecture electrodes for MSCs have been 3D‐printed.^[^ ^93^, ^94^, ^95^, ^96^, ^97^, ^98^ ^]^ Using a graphene oxide (GO) suspension and hydroxypropyl methylcellulose mixture as the “ink,” Yao et al. printed 3D GO structures with multiple orthogonal layers of parallel porous cylindrical rods.^[^ ^83^ ^]^ Freeze‐drying and thermal annealing were then performed to obtain a 3D graphene (G) aerogel scaffold, which was utilized as a current collector for MnO_2_ deposition (**Figure** **7a**,b). Besides simultaneously enabling efficient electron transport and ion diffusion, an ultra‐high MnO_2_ loading of 182.2 mg cm^–2^ was obtained for a 4‐mm‐thick 3D G aerogel/MnO_2_ electrode, which then exhibited a record‐high areal capacitance of 44.13 F cm^–2^ (Figure 7c). Symmetric MSCs consisting of two 4‐mm‐thick 3D G aerogel/MnO_2_ electrodes delivered a maximum areal energy density of 1.56 mWh cm^–2^ and retained 92.9% of their initial capacitance after 20 000 cycles.

In another study, atomically thin (1–3 nm) 2D titanium carbide (Ti_3_C_2_T*_x_*) transition‐metal carbide, carbonitride, and nitride (MXene) nanosheets, which are promising electrochemically active materials for supercapacitors, were fabricated into aqueous printable inks for 3D printing.^[^ ^84^ ^]^ Freestanding Ti_3_C_2_T*_x_* microlattices were directly printed onto interdigitated electrodes on the substrate to form current‐collector‐free, in‐plane, symmetric MSCs following coating with solid‐state electrolytes (Figure 7d,e). The Ti_3_C_2_T*_x_* mass loading was 8.5 mg cm^–2^, yielding a high areal capacitance of 2.1 F cm^–2^. The corresponding Ragone plot revealed the considerably higher areal energy and power densities of the 3D‐imprinted Ti_3_C_2_T*_x_* MSCs compared to Ti_3_C_2_T*_x_*‐based MSCs fabricated using other techniques (Figure 7f).

Also using 3D printing, Li et al. fabricated stretchable MSCs with high areal energy and power densities.^[^ ^85^ ^]^ Using a viscous pseudoplastic nanocomposite ink composed of Ti_3_C_2_T*_x_* MXene nanosheets, MnO_2_ nanowires, silver nanowires, and fullerene, those researchers printed thick interdigitated electrodes possessing a honeycomb‐like microporous scaffold in combination with a layered cell wall structure on a flexible substrate (Figure 7g,h). When the 3D‐printed electrodes were stretched in the strain direction, the honeycomb‐like micropores and layer slippage of the layered cell wall improved the MSC structural stability and prevented energy‐storage deterioration. Even with stretching from 0% to 50%, the MSC capacitance remained above 80% (Figure 7i). Thus, 3D‐printed MSCs outperform most reported stretchable MSCs in terms of areal energy and power density (19.2 μWh cm^–2^ and 58.3 mW cm^–2^, respectively), because of the unique structure of the 3D‐printed electrodes.

The above examples indicate that 3D printing can be used to fabricate 3D architecture electrodes with considerably higher mass loading of electrochemically active materials and higher mechanical stability than electrodes fabricated using alternative methods.

## 5 Challenges and Perspectives

**Figure 8:**
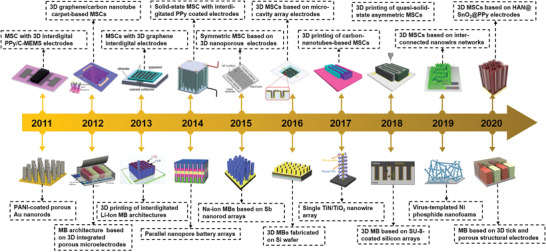
Brief development timeline featuring representative 3D architecture electrodes for MBs and MSCs spanning the past decade. Inset images: MSC with 3D interdigital PPy/C‐MEMS electrodes (Reproduced with permission.^[^ ^72^ ^]^ Copyright 2011, Elsevier); 3D G/carbon nanotube carpet‐based MSCs (Reproduced with permission.^[^ ^73^ ^]^ Copyright 2012, American Chemical Society); MSCs with 3D G interdigital electrodes (Reproduced with permission.^[^ ^99^ ^]^ Copyright 2013, The Electrochemical Society); Solid‐state MSC with interdigitated PPy‐coated electrodes (Reproduced with permission.^[^ ^100^ ^]^ Copyright 2014, Elsevier); Symmetric MSC based on 3D nanoporous electrodes (Reproduced with permission.^[^ ^101^ ^]^ Copyright 2015, Wiley‐VCH); 3D MSCs based on microcavity array electrodes (Reproduced with permission.^[^ ^102^ ^]^ Copyright 2016, American Chemical Society); 3D printing of carbon‐nanotube‐based MSCs (Reproduced with permission.^[^ ^103^ ^]^ Copyright 2017, American Chemical Society); 3D printing of quasi‐solid‐state asymmetric MSCs (Reproduced with permission.^[^ ^77^ ^]^ Copyright 2018, Wiley‐VCH); 3D MSCs based on inter‐connected nanowire networks (Reproduced with permission.^[^ ^104^ ^]^ Copyright 2019, Elsevier); 3D MSCs based on HAN@SnO_2_@PPy electrodes (Reproduced with permission.^[^ ^81^ ^]^ Copyright 2020, Nature Publishing Group); PANI‐coated porous Au nanorods (Reproduced with permission.^[^ ^46^ ^]^ Copyright 2011, American Chemical Society); MB architecture based on 3D integrated porous microelectrodes (Reproduced with permission.^[^ ^62^ ^]^ Copyright 2012, Nature Publishing Group); 3D printing of interdigitated Li‐ion MB architectures (Reproduced with permission.^[^ ^105^ ^]^ Copyright 2013, Wiley‐VCH); Parallel nanopore battery arrays (Reproduced with permission.^[^ ^106^ ^]^ Copyright 2014, Nature Publishing Group); Na‐ion MBs based on Sb nanorod arrays (Reproduced with permission.^[^ ^107^ ^]^ Copyright 2015, The Royal Society of Chemistry); 3D MBs fabricated on Si wafer (Reproduced with permission.^[^ ^55^ ^]^ Copyright 2016, Wiley‐VCH); Single TiN/TiO_2_ nanowire array (Reproduced with permission.^[^ ^65^ ^]^ Copyright 2017, Elsevier); 3D MB based on SU‐8‐coated silicon arrays (Reproduced with permission.^[^ ^61^ ^]^ Copyright 2018, Elsevier); Virus‐templated Ni phosphide nanofoams (Reproduced with permission.^[^ ^108^ ^]^ Copyright 2019, Wiley‐VCH); MB based on 3D thick and porous structural electrodes (Reproduced with permission.^[^ ^64^ ^]^ Copyright 2020, Wiley‐VCH).

**Figure** **8** briefly outlines the development of 3D architecture electrodes for MBs and MSCs over the past decade, highlighting some representative examples. Various 3D architecture electrodes with different morphologies and materials have been designed, fabricated, and investigated with the aim of achieving improved energy storage and delivery performance within a limited footprint area. **Tables** **1** and **2** also summarize representative 3D architecture electrodes for MBs and MSCs developed in the past decade, with details of the electrochemically active materials, electrode structure, electrolytes, capacity/capacitance, energy density, power density, and lifespan. Unlike 2D thin‐/thick‐film electrodes, which require an inevitable trade‐off between the attainable energy and power, these 3D architecture electrodes guarantee simultaneous enhancement of both the energy and power densities while simultaneously ensuring longer cycle life. Thus, it can be concluded that the 3D architecture electrode design is conducive to synergistic performance improvement of MBs and MSCs. Even though the performance of the MBs and MSCs discussed in this review has already surpassed that of many commercial MBs and MSCs, successful transitioning of MBs and MSCs based on 3D architecture electrodes from lab to market remains an arduous task. A series of challenges must be overcome, that is, further performance optimization, scalable and cost‐effective production, reliable device encapsulation, and compatible integration with IoT devices must be realized.

**Table 1:** Summary of recently reported MBs based on 3D architecture electrodes and their electrochemical performance

Electrode materials	Electrode structure	Electrolyte	Capacity	Energy density	Power density	Lifespan	Ref.
TiO_2_ // Li	Nanorods	LiClO_4_/PC	0.0112 mAh cm^–2^	–	–	40% (50)	^[^ ^45^ ^]^
PANI // Li	Porous nanorods	LiClO_4_/EC/DMC	32 μAh cm^–2^	–	–	64% (75)	^[^ ^46^ ^]^
Li // LiCoO_2_	Nanorods	LiPF_6_/EC/DMC	0.102 mAh cm^–2^	–	–	83% (100)	^[^ ^47^ ^]^
TiO_2_ // Li	Nanowires	LiPF_6_/EC/DMC	0.032 mAh cm^–2^	–	–	100% (600)	^[^ ^48^ ^]^
RuO*_x_* N*_y_* S*_z_* // Li	Porous structure	LiPF_6_/EC/DMC	5 mAh cm^–2^	–	–	50% (2600)	^[^ ^49^ ^]^
Ge // Li	Nanonets	LiPF_6_/EC/PC/DEC	2 mAh cm^–2^	–	–	99.4% (140)	^[^ ^50^ ^]^
MnO_2_ // Li	Porous structure	LiClO_4_/EC/DMC	–	45.5 mWh cm^–2^ μm^–1^	5300 μW cm^–2^ μm^–1^	–	^[^ ^52^ ^]^
TiO_2_ // Li	Nanotubes	LiTFSI/EC/DEC	370 μAh cm^–2^	–	–	100% (40)	^[^ ^55^ ^]^
TiO_2_ // Li	Nanopillars	LiClO_4_/PC	≈210 μAh cm^–2^ μm^–1^	–	≈115 μW cm^–2^	≈100% (400)	^[^ ^56^ ^]^
a‐VO_2_ // Li	Nanorods	LiClO_4_/PC	130 μAh cm^–2^	–	–	85.2% (50)	^[^ ^57^ ^]^
Lithiated Si // LiNi_0.8_Co_0.15_Al_0.05_O_2_	Micropillars	LiClO_4_/PC	1.8 mAh cm^–2^	5.2 mWh cm^–2^	–	92% (100)	^[^ ^61^ ^]^
Ni—Sn // Li*_x_* MnO_2_	Porous structure	LiClO_4_/EC/DEC	–	15 mWh cm^–2^ μm^–1^	7.4 mW cm^–3^ μm^–1^	64% (15)	^[^ ^62^ ^]^
Ni—Sn // Li*_x_* MnO_2_	Porous structure	LiClO_4_/EC/DEC	–	6.5 μWh cm^–2^ μm^–1^	3600 µW cm^–2^ μm^–1^	80% (100)	^[^ ^63^ ^]^
V_2_O_5_ // Li	Porous structure	PEO/LiTFSI/DOL/DME	–	1.242 J cm^–2^	75.5 mW cm^–2^	75% (200)	^[^ ^64^ ^]^
TiO_2_ // Li	Nanowires	LiPF_6_/EC/DEC	0.146 mAh cm^–2^	–	–	82% (1000)	^[^ ^65^ ^]^
Ni(OH)_2_ // Zn	Porous structure	1 M KOH	–	0.26 mW h cm^–2^	33.8 mW cm^–2^	75.8% (2200)	^[^ ^66^ ^]^
Li // LiFePO_4_	Nanowires	LiPF_6_/EC/DEC	152 μAh cm^–2^	–	–	93.7% (450)	^[^ ^67^ ^]^
Li_4_Ti_5_O_12_ // LiFePO_4_	Multilayers	LiClO_4_/EC/DEC	–	9.7 J cm^–2^	2.7 mW cm^–2^	≈98% (30)	^[^ ^105^ ^]^
V_2_O_5_ // V_2_O_5_	Porous structure	LiPF_6_EC/DEC	–	0.6 μWh cm^–2^ μm^–1^	0.49 μW cm^–2^ μm^–1^	81% (1000)	^[^ ^106^ ^]^
Ni_5_P_4_ // Li	Nanofoams	LiPF_6_/EC/DEC	677 mAh cm^–3^	–	–	80% (100)	^[^ ^108^ ^]^

**Table 2:** Summary of recently reported MSCs based on 3D architecture electrodes and their electrochemical performance

Electrode materials	Electrode structure	Electrolyte	Capacitance	Energy density	Power density	Lifespan	Ref.
PPy/C‐MEMS // PPy/C‐MEMS	Nanopillars	0.1 m KCl	78.35 ± 5.67 mF cm^−2^	–	0.63 ± 0.04 mW cm^−2^	56% (1000)	^[^ ^72^ ^]^
G/CNTCs // G/CNTCs	Nanopillars	1 m BMIM‐BF_4_	3.93 mF cm^–2^	2.42 mWh cm^–3^	135 W cm^–3^	98.4% (8000)	^[^ ^73^ ^]^
RuO_2_ // RuO_2_	Porous structure	PVA/H_3_PO_4_/SiW_a_	–	0.126 mWh cm^–2^	7.9 mW cm^–2^	95% (2000)	^[^ ^74^ ^]^
LSG/CoNi_2_S_4_ // LSG	Nanosheets	PVA/KOH	122.4 F cm^–3^	49 W h L^–1^	–	93.9% (10 000)	^[^ ^75^ ^]^
Co_3_O_4_/Pt // Co_3_O_4_/Pt	Nanonetworks	1 m KOH	31.7 F cm^–3^	3.17 mWh cm^–3^	–	91.9% (5000)	^[^ ^76^ ^]^
VO*_x_*/rGO //G‐VNQDs/rGO	Multilayers	LiCl/PVA	207.9 mF cm^–2^	73.9 μWh cm^–2^	–	65% (8000)	^[^ ^77^ ^]^
ITO NWs@MnO_2_ // ITO NWs@MnO_2_	Nanowires	LiCl/PVA	193.8 mF cm^–2^	26.94 mWh cm^–2^	15.07 mW cm^–2^	61.1% (20 000)	^[^ ^78^ ^]^
LIG/PPy // LIG/PPy	Porous structure	PVA/H_2_SO_4_	2412.2 mF cm^–2^	134.4 μWh cm^–2^	325 μW cm^–2^	95.6% (10 000)	^[^ ^79^ ^]^
Cu(OH)_2_@FeOOH/Cu //Cu(OH)_2_@FeOOH /Cu	Nanotubes	NaOH/(NH_4_)_2_SO_3_	58.0 mF cm^–2^	–	18.07 μW cm^–2^	97% (10 000)	^[^ ^80^ ^]^
HAN@SnO_2_@MnO_2_ //HAN@SnO_2_@PPy	Honeycomb‐like porous structure	1 m Na_2_SO_4_	128 mF cm^–2^	160 μWh cm^–2^	40 mW cm^–2^	≈100% (10 000)	^[^ ^81^ ^]^
MnO_2_ // MnO_2_ In‐parallel	Microtubes	5 m LiNO_3_	0.75 F cm^–2^	0.1 mW h cm^–2^	0.16 mW cm^–2^	82% (10 000)	^[^ ^82^ ^]^
MnO_2_ // MnO_2_ Integrated	Microtubes	5 m LiNO_3_	0.39 F cm^–2^	0.06 mW h cm^–2^	0.2 mW cm^–2^	84% (10 000)	^[^ ^82^ ^]^
G/MnO_2_ // G/MnO_2_	Multilayers	3 m LiCl	18.74 F cm^–2^	1.56 mW h cm^–2^	–	92.9% (20 000)	^[^ ^83^ ^]^
Ti_3_C_2_T*_x_* // Ti_3_C_2_T*_x_*	Multilayers	PVA/H_2_SO_4_	2.1 F cm^–2^	24.4 μWh cm^–2^	0.64 mW cm^–2^	90% (10 000)	^[^ ^84^ ^]^
Ti_3_C_2_T*_x_*/Ag // MnO/C60	Multilayers	PVA/KOH	216.2 mF cm^–2^	19.2 μWh cm^–2^	58.3 mW cm^–2^	85% (10 000)	^[^ ^85^ ^]^
Ti_3_C_2_T*_x_* // Ti_3_C_2_T*_x_*	Multilayers	PVA/H_2_SO_4_	56.8 mF cm^–2^	0.63 μWh cm^–2^	0.33 mW cm^–2^	–	^[^ ^96^ ^]^
Au/ δ‐MnO_2_ // Au/δ‐MnO_2_	Porous structure	1 m Na_2_SO_4_	≈922 mF cm^–3^	–	≈295 W cm^–3^	88% (20 000)	^[^ ^101^ ^]^
PANI // PANI	Nanofibers	PVA/H_2_SO_4_	65.1 mF cm^–2^	0.011 mWh cm^–2^	–	85.7% (1000)	^[^ ^102^ ^]^
3D CNTs // 3D CNTs	Multilayers	PVA/H_3_PO_4_	2.44 F cm^–2^	0.12 mWh cm^–3^	3.72 W cm^–3^	93% (1500)	^[^ ^103^ ^]^

**Figure 9:**
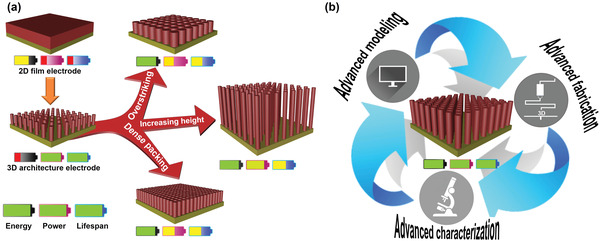
a) Schematic illustration of three methods of increasing the mass loading of 3D architecture electrodes, taking nanorod arrays as an example. b) Development overview of 3D architecture electrodes with high energy density, high power density, and long lifespan through integration of advanced fabrication, advanced characterization, and advanced modeling techniques.

Most current research aims to foster strengths and circumvent the weaknesses of 3D architecture electrodes toward further performance improvement of MBs and MSCs. As mentioned above, the sufficient empty voids in 3D architecture electrodes enable formation of interconnected electrolyte‐filled networks to facilitate ion transport, thereby improving the power density. These voids also provide sufficient space to accommodate the volume changes of the electrochemically active materials during charging–discharging, thereby enhancing the cyclability. However, these empty voids are a negative factor as regards increased mass loading of electrochemically active materials, and this is undesirable in the context of energy density enhancement. As illustrated in **Figure** **9a**, in which nanorod arrays are considered as an example of 3D architecture electrodes, there are three main methods of improving the mass loading: increasing the diameter, increasing the height, or employing dense packing. However, each method has weaknesses. Both an increased diameter and dense packing reduce the spaces between the structural units; this adversely affects the ion transport efficiency, particularly at higher charging–discharging rates, with the limited inner spaces being unable to accommodate the volume changes, yielding poor electrode cyclability.^[^ ^36^, ^109^ ^]^ In addition, increased height causes collapse and/or agglomeration of structural units, which further deteriorates the ion transport and cyclability.^[^ ^107^, ^110^ ^]^ Moreover, these three methods significantly increase the electrode specific surface area; thus, they may exacerbate the side reactions and cause higher electrolyte consumption. Electrolyte engineering and artificial solid electrolyte interphase (SEI) layer construction are two effective strategies to suppress unfavorable side reactions and avoid excessive electrolyte consumption.^[^ ^111^, ^112^, ^113^, ^114^ ^]^ The same problems arise for micro/nanoporous structures used as 3D architecture electrodes, as these issues are closely related to pore size, pore distribution, and pore wall thickness. Therefore, a comprehensive strategy must be developed by integrating advanced fabrication, characterization, and modeling techniques to rationally design and fabricate 3D architecture electrodes, so as to realize high energy density, high power density, and long lifespan (Figure 9b). These aspects are discussed individually below. i) Advanced fabrication techniques should enable cost‐effective production of pre‐designed 3D architecture electrodes with optimized structural parameters and morphologies. To this end, 3D printing holds great promise; however, there is a lack of suitable inks composed of conductive materials and/or electrochemically active materials.^[^ ^115^, ^116^ ^]^ To date, only a few emulsion inks containing conductive emulsion inks for 3D printing of 3D architecture electrodes have been prepared. Therefore, considerable research effort should be devoted to developing such emulsion inks. Moreover, improved printing resolution is another key target for the future development of 3D printing; this advance would allow printing of 3D architecture electrodes with more precise and complex structures.^[^ ^117^, ^118^ ^]^

ii) Advanced characterization techniques, especially in situ characterization methods (including in situ transmission electron microscopy, in situ Raman spectroscopy, in situ nuclear magnetic resonance, combined time‐resolved X‐ray diffraction and mass spectroscopy, and in situ synchrotron X‐ray characterization) are expected to provide detailed information on 3D architecture electrodes during charging–discharging (e.g., their morphology, structure, and component evolution/degradation). Such information will be highly valuable as it will reveal the evolution of 3D architecture electrodes during electrochemical processes and elucidate their electrode degradation mechanisms.^[^ ^119^, ^120^, ^121^, ^122^, ^123^, ^124^, ^125^ ^]^ Thus, these advanced characterization results will guide further optimization of 3D architecture electrodes. Currently, a single structural unit of a 3D architecture electrode is typically selected as the research subject of an in situ microscopy characterization. In the future, compatible electrochemical cells should be designed and constructed to enable in situ characterization of all 3D architecture electrodes rather than a single unit.

iii) Advanced modeling based on physical and chemical principles, such as, concentrated solution theory, the Butler–Volmer equation, porous electrode theory, Ohm's law, and finite element analysis, could simulate the mass transport behavior of ions and electrons within a confined space.^[^ ^126^, ^127^, ^128^ ^]^ Theoretical simulation and modeling is expected to provide direct insights into the current/voltage distribution, active material utilization, and electrolyte concentration gradients of 3D architecture electrodes.^[^ ^22^, ^129^, ^130^ ^]^ These insights will help identify the electrochemical processes occurring within the electrodes and electrolytes during the charge and discharge cycles. Improved understanding of the electrochemical reaction kinetics during charging–discharging could allow clear definition of the decisive factors influencing the various physical processes within microscale 3D architecture electrodes. Moreover, combined advanced characterization and advanced modeling results could allow strategic optimization of the structural parameters and morphologies of 3D architecture electrodes to achieve optimal MB and MSC performance.

Additionally, the wettability of 3D architecture electrodes with solid‐state electrolytes should attract particular focus, as all‐solid‐state MBs and MSCs are preferable for IoT‐based applications. Incomplete filling of 3D architecture electrodes with solid‐state electrolytes not only reduces the utilization efficiency of the electrochemically active materials, but also adversely impacts the electrode lifespan and safety.^[^ ^131^, ^132^, ^133^, ^134^ ^]^ For MBs in particular, incomplete filling of electrolytes causes formation of a non‐uniform SEI layer in the 3D architecture electrodes, which further induces electrolyte consumption, reduced Coulombic efficiency, or metal dendrite formation. Therefore, 3D architecture electrodes should have sufficiently high wettability to ensure complete filling of solid‐state electrolytes in all electrode voids. As regards the filling methods, electrolytes usually intrude into electrode voids via diffusion and capillary forces. Sometimes, application of an evacuation‐generated negative pressure is necessary to facilitate electrolyte filling. However, complete electrolyte filling of all voids of a 3D architecture electrode remains challenging. In contrast, the ALD technique has been shown to be a powerful method for complete and uniform electrolyte filling, even for 3D architecture electrodes with complex porous structures.^[^ ^135^, ^136^, ^137^ ^]^ Unfortunately, only a few solid‐state electrolytes suitable for deposition via the ALD technique are currently available; hence, development of novel ALD precursors for solid‐state electrolytes should also be a key research focus.

Finally, the issues of mass production, reliable device packaging, and integration compatibility of MBs and MSCs based on 3D architecture electrodes should also attract considerable research attention in the near future.

Overall, we hope this review will draw more attention to 3D architecture electrode design and fabrication, thereby stimulating continuous innovations in this field and advancing the development of MBs and MSCs with high energy density, high power density, and long lifespan. It can be expected that MBs and MSCs based on 3D architecture electrodes will satisfy the energy requirements of IoT devices in the near future.

## Conflict of Interest

The authors declare no conflict of interest.
